# Development of Differentiating Infected from Vaccinated Animals (DIVA) Real-Time PCR for African Horse Sickness Virus Serotype 1 

**DOI:** 10.3201/eid2812.220594

**Published:** 2022-12

**Authors:** Yifan Wang, Jasmine Ong, Oi Wing Ng, Tapanut Songkasupa, Eileen Y. Koh, Jeslyn P.S. Wong, Kanokwan Puangjinda, Charlene Judith Fernandez, Taoqi Huangfu, Lee Ching Ng, Siow Foong Chang, Him Hoo Yap

**Affiliations:** Animal and Veterinary Service, National Parks Board, Singapore (Y. Wang, J. Ong, O.W. Ng, E.Y. Koh, C.J. Fernandez, T. Huangfu, S.F. Chang, H.H. Yap);; National Institute of Animal Health, Bangkok, Thailand (T. Songkasupa, K. Puangjinda);; Environmental Health Institute, National Environment Agency, Singapore (J.P.S. Wong, L.C. Ng)

**Keywords:** African horse sickness virus, AHSV-1, virology, DIVA assay, viruses, vector-borne infections, Thailand, Differentiating Infected from Vaccinated Animals

## Abstract

African horse sickness (AHS) is a highly infectious and often fatal disease caused by 9 serotypes of the orbivirus African horse sickness virus (AHSV). In March 2020, an AHS outbreak was reported in Thailand in which AHSV serotype 1 was identified as the causative agent. Trivalent live attenuated vaccines serotype 1, 3, and 4 were used in a targeted vaccination campaign within a 50-km radius surrounding the infected cases, which promptly controlled the spread of the disease. However, AHS-like symptoms in vaccinated horses required laboratory diagnostic methods to differentiate infected horses from vaccinated horses, especially for postvaccination surveillance. We describe a real-time reverse transcription PCR–based assay for rapid characterization of the affecting field strain. The development and validation of this assay should imbue confidence in differentiating AHS-vaccinated horses from nonvaccinated horses. This method should be applied to determining the epidemiology of AHSV in future outbreaks.

African horse sickness (AHS) is a fatal vector-borne disease affecting all species of equids. The disease has major economic consequences for the equine industry. The World Organisation for Animal Health (WOAH) lists AHS as a notifiable disease, which affects the movement of horses to and from affected areas (*1*). The disease is transmitted through biting midges of the *Culicoides* genus; the 2 species *C. imicola* and *C. bolitinos* are considered the most critical vectors of AHSV (*2*,*3*). AHS is known to be endemic to large areas of sub-Saharan Africa and to have spread to Morocco, the Middle East, India, and Pakistan (*4*,*5*). More recently, outbreaks were reported in the Iberian Peninsula, Thailand, and Malaysia (*6*–*10*).

AHS is caused by the AHS virus (AHSV), which belongs to the genus *Orbivirus* (family Reoviridae). The AHSV genome consists of 10 double-stranded RNA segments encoding 7 structural proteins (viral protein [VP] 1–7) and >3 nonstructural proteins (NS1–NS3) (*11*,*12*). To date, 9 known serotypes of AHSV (AHSV-1–9) have been determined by virus neutralization. Some evidence exists of serologic cross-reactions among serotypes 1 and 2, 3 and 7, 5 and 8, and 6 and 9 (*13*–*15*).

Currently no specific treatment for AHS exists aside from rest, supportive treatment, and care. Vaccination of at-risk equids remains the most effective way to prevent and control the disease (*16*). Live attenuated vaccines (LAV), which provide broad protection against all 9 AHSV serotypes, are produced by Onderstepoort Biologic Products (OBP; https://www.obpvaccines.co.za) and are commercially available. The vaccine is supplied as 2 polyvalent vials: trivalent, containing AHSV-1, AHSV-3, and AHSV-4; and tetravalent, containing AHSV-2, AHSV-6, AHSV-7, and AHSV-8 (*14*,*17*,*18*).

In March 2020, an AHS outbreak was reported in Nakhon Ratchasima Province in Thailand; 610 horses were affected, and the case-fatality rate was ≈93% (*7*,*19*). Samples were submitted to the National Institute of Animal Health, Thailand, and the Centre for Animal and Veterinary Sciences (CAVS) of the National Parks Board Singapore for laboratory investigation. At CAVS, full AHSV genome sequence was obtained by using Oxford Nanopore Sequencing, and AHSV-1 virus was identified to be the strain responsible for this outbreak (*9*). Subsequently, a targeted vaccination campaign using the trivalent OBP vaccine was conducted in Thailand within a 50-km radius of the infected cases.

To distinguish infected equid from vaccinated equid, having a Differentiating Infected from Vaccinated Animals (DIVA) strategy in place becomes critical for detecting disease early, limiting the movement of at-risk equids, and enabling the authorities to better deal with the biosecurity threat posed by the virus in the outbreak. Sequence analysis of the full VP2 gene was performed for DIVA, and differences in the following amino acid positions could be identified between the outbreak strain (GenBank accession no. QM158105.1) and the OBP LAV strain (GenBank accession no. AKP20114). These 10 mutation occurrences—K357N, I383V, K522R, K580R, I587T, I588V, T660I, Y803N, T889M, and T910A—were observed in the wild-type AHS circulating in Thailand. These variants arose from mutations originating from wild-type and attenuated AHSV, which resulted in pathogenicity and severity of disease as demonstrated by reported symptoms or duration of disease in individual animals. However, sequencing of the full VP2 gene is time-consuming and hampered by the small amount of AHSV RNA in samples. We report the design and validation of a sensitive and rapid real-time reverse transcription PCR (rRT-PCR) that can differentiate the AHSV-1 outbreak strain in Thailand from the strain used in the vaccination campaign. This assay is designed to strengthen AHS surveillance programs and outbreak management by enabling confident and rapid detection of infected horses and better understanding of vaccine breakthrough, if it occurs.

## Materials and Methods

### Horse Samples and Vaccine Samples

Tissue homogenates (which include lung, spleen, and heart) and blood samples from horses exhibiting clinical signs of AHS were submitted to the National Institute of Animal Health, Thailand. Samples were collected from western (Prachuap Khiri Khan, Phetchaburi, and Ratchaburi), northeastern (Nakhon Ratchasima), and eastern (Chon Buri) provinces in Thailand. A total of 3 tissue homogenate samples and 4 blood samples from this initial batch of samples were received at CAVS for laboratory analysis (Table 1) (*9*). Separately, AHSV-1 OBP vaccine samples were obtained from the European Union Reference Laboratory for African horse sickness and Bluetongue, Central Veterinary Laboratory (Animal Health) in Madrid, Spain (Table 1). Samples from the initial batch cases were subsequently included for evaluation of the AHSV-1 DIVA assay. To further validate the DIVA assays designed in this study, whole blood preserved in EDTA and tissue homogenates were collected from clinically affected horses (n = 31) and vaccinated horses (n = 12) from the various provinces in Thailand (Table 1).

**Table 1:** Horse samples and vaccine samples from Thailand used in characterization of affecting strain of AHSV by DIVA rRT-PCR*

No.	Sample ID	Sample type	Source	
Initial batch of samples (n = 7) reported in (*9*)			
1	110983/63	Tissue homogenate	Pak Chong, Nakhon-Ratchasima	
2	111495/63	Whole blood	Cha-am, Phetchaburi	
3	111406/63	Tissue homogenate	Ko Chan, Chonburi	
4	111146/63	Whole blood	Pak Chong, Nakhon-Ratchasima	
5	112080/63	Whole blood	Hua Hin, Prachuap Khiri Khan	
6	111789/63	Whole blood	Damnoen Saduak, Ratchaburi	
7	111367/63	Tissue homogenate	Hua Hin, Prachuap Khiri Khan	
8	AHSV-1 OBP VACCINE 10^−3^	vaccine	EURL (Spain)	
9	AHSV-1 OBP VACCINE 10^−4^	vaccine	EURL (Spain)	
10	AHSV-1 OBP VACCINE 10^−5^	vaccine	EURL (Spain)	
Samples collected from clinically affected horses (n = 31) in this study				
1	111146/63	Whole blood	Nakhon-Ratchasima	
2	111147/63-5	Whole blood	Nakhon-Ratchasima	
3	111147/63-19	Whole blood	Nakhon-Ratchasima	
4	111147/63-20	Whole blood	Nakhon-Ratchasima	
5	111147/63-21	Whole blood	Nakhon-Ratchasima	
6	111147/63–22	Whole blood	Nakhon-Ratchasima	
7	111162/63-A	Whole blood	Nakhon-Ratchasima	
8	111162/63-B	Whole blood	Nakhon-Ratchasima	
9	111164/63-1	Whole blood	Nakhon-Ratchasima	
10	111164/63-4	Whole blood	Nakhon-Ratchasima	
11	111367/63-B	Tissue homogenate	Prachuap Khiri Khan	
12	111406/63-A	Tissue homogenate	Chonburi	
13	111406/63-B	Tissue homogenate	Chonburi	
14	111496/63	Whole blood	Prachuap Khiri Khan	
15	111790/63	Whole blood	Phetchaburi	
16	112080/63	Whole blood	Prachuap Khiri Khan	
17	112590/63	Whole blood	Phetchaburi	
18	112594/63	Whole blood	Phetchaburi	
19	112680/63	Whole blood	Phetchaburi	
20	113308/63	Whole blood	Srakaew	
21	113480/63	Whole blood	Prachuap Khiri Khan	
22	113481/63	Whole blood	Prachuap Khiri Khan	
23	113489/63	Whole blood	Srakaew	
24	113561/63-1	Whole blood	Phetchaburi	
25	113561/63-3	Whole blood	Phetchaburi	
26	113869/63	Whole blood	Prachuap Khiri Khan	
27	113870/63-11	Whole blood	Prachuap Khiri Khan	
28	113870/63-5	Whole blood	Phetchaburi	
29	113871/63-N	Whole blood	Phetchaburi	
30	113871/63-5140	Whole blood	Phetchaburi	
31	113908/63	Whole blood	Phetchaburi	
Samples collected from vaccinated horses (n = 12) in this study				
1	116187/63	Whole blood	Nakhon-Ratchasima	
2	122045/63	Whole blood	Pathumthani	
3	135560/63	Tissue homogenate	Lopburi	
4	137720/63	Tissue homogenate	Rayong	
5	118696/63	Whole blood	Srakaew	
6	120865/63	Whole blood	Ratchaburi	
7	136979/63	Whole blood	Suphanburi	
8	121673/63-A	Whole blood	Prachuap Khiri Khan	
9	121673/63-B	Whole blood	Prachuap Khiri Khan	
10	121673/63-C	Whole blood	Prachuap Khiri Khan	
11	124916/63-A	Whole blood	Prachuap Khiri Khan	
12	124916/63-B	Whole blood	Prachuap Khiri Khan	

*AHSV, African horse sickness virus; DIVA, Differentiating Infected from Vaccinated Animals; ID, identification; rRT-PCR, real-time reverse transcription PCR.

### Detection and Serotyping of AHSV Using rRT-PCR

We performed RNA extraction by using the MagMax Pathogen RNA/DNA kit (ThermoFisher Scientific, https://www.thermofisher.com). In brief, 100 µL of the tissue homogenate, whole blood, and vaccine samples underwent nucleic acid extraction according to manufacturer recommendations. We eluted the extracted RNA in 90 µL elution buffer and performed an rRT-PCR targeting the AHSV VP7 gene to detect the presence of AHSV (*20*,*21*). Samples for which AHSV was detected were further characterized by a serotyping rRT-PCR targeting the AHSV VP2 gene (*22*). We used a volume of 2.5 µL of extracted RNA in both rRT-PCRs and subsequently used those confirmed to be AHSV-1 to validate the AHSV-1 DIVA assay.

### AHSV-1 DIVA rRT-PCR Development

#### Primer and Probe

**Figure 1:**
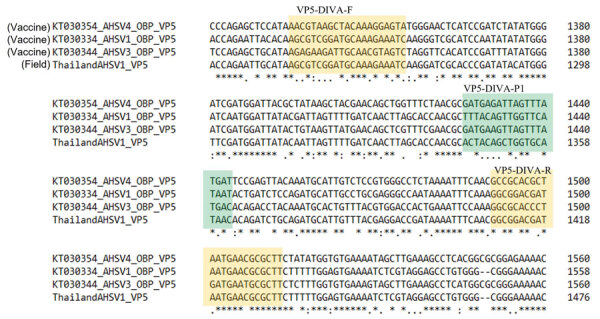
Nucleotide sequence alignment of the VP5 gene for the OBP AHSV vaccine strains and Thailand AHSV-1 field isolate at the 1321–1560 region (numbering according to the OBP AHSV-1 isolate, GenBank accession no. KT030334) by multiple sequence alignment tool in Clustal Omega (https://www.ebi.ac.uk/Tools/msa/clustalo). OBP strain GenBank accession nos.: AHSV-1, KT030334; AHSV-3, KT030344; AHSV-4, KT030354. Thailand AHSV-1 isolate GenBank accession no.: MT711962. Yellow indicates the primer-binding regions (VP5-DIVA-F/R) and green the probe-binding region (VP5-DIVA-P1). AHSV, African horse sickness virus; DIVA, Differentiating Infected from Vaccinated Animals; OBP, Onderstepoort Biologic Products (https://www.obpvaccines.co.za); VP, viral protein.

In a previous study (*9*), full-length genome segments of the AHSV-1 strain in Thailand were obtained by using the Single Primer Amplification approach (*23*,*24*), followed by Oxford Nanopore sequencing (GenBank accession nos. MT711958–67). Compared with the sequence of AHSV-1 strain (1/Labstr/ZAF/1998/OBP-116) used for production of the LAV-OBP vaccine (GenBank Accession nos. KT030330.1–9.1) (*25*), a region near the 3′ end of the VP5 gene shared lower sequence similarity (Figure 1) and was thus exploited for primer and probe design. The VP5 protein is not involved in AHSV antibody–mediated neutralization tests, as compared to the widely used but more conserved VP7 protein (*12*). We then designed primers and probes by Primer3web version 4.1.0 (https://primer3.ut.ee) and further optimized them manually (Table 2).

**Table 2:** Sequence and position of the AHSV-1 DIVA primer and probes used in study of development of PCR to characterize affecting strain of AHSV, Thailand*

Primer/probe	Sequence, 5′ → 3′	Sense	Position
VP5-DIVA-F	5′ AGCGTCGGATGCAAAGAAATC 3′	+	1335–1355
VP5-DIVA-P1	5′ FAM- ACTACAGCTGGTGCATAAC 3′ MGB	+	1426–1444
VP5-DIVA-P2-vac	5′ FAM- TTTACAGTTGGTTCATAAT 3′ MGB	+	1426–1444
VP5-DIVA-R	5′ AAGCGCGTTCATTATCGTCC 3′	–	1494–1513

*Position numbering is with reference to AHSV-1 (GenBank accession no. KT030334). AHSV, African horse sickness virus; DIVA, Differentiating Infected from Vaccinated Animals.

We used 2 programs to assess the newly designed primers’ and probes’ annealing specificity: Primer-BLAST (https://www.ncbi.nlm.nih.gov/tools/primer-blast) and BLAST Global Alignment (https://blast.ncbi.nlm.nih.gov/Blast.cgi). Primer-BLAST determined whether the primers were specific to the AHSV-1 strain and not the other 2 strains in the trivalent vaccine dose, whereas BLAST Global Alignment aligned the AHSV vaccines and wild-type nucleotide sequences with probes by Needleman-Wunsch algorithm to ensure differentiation between vaccine and outbreak strain (Appendix). 

#### rRT-PCR

We designed 2 rRT-PCRs using different probes to target the same region of VP5 gene (1335–1513, numbering according to KT030334) of AHSV-1. Probe VP5-DIVA-P1 specifically targets the outbreak strain, whereas probe VP5-DIVA-P2-vac targets the LAV-OBP vaccine strain. We conducted the rRT-PCR in a 25-µL reaction mixture containing 12.5 µL of 2 × RT-PCR buffer (AgPath-ID One-Step RT-PCR; ThermoFisher Scientific), 1 µL of 25 × RT-PCR enzyme mix, 0.5 µL (0.2 µmol) each of forward (VP5-DIVA-F: 5′-AGCGTCGGATGCAAAGAAATC-3′) and reverse (VP5-DIVA-R: 5′-AAGCGCGTTCATTATCGTCC-3′) primers (10 µmol/L), 0.5 µL (0.12 µmol) of probe (6 µmol/L) (AHSV-1 DIVA rRT-PCR1: VP5-DIVA-P1 5′-FAM- ACTACAGCTGGTGCATAAC-3′ MGB or AHSV-1 DIVA rRT-PCR2: VP5-DIVA-P2-vac 5′ FAM- TTTACAGTTGGTTCATAAT 3′ MGB), 7.5 µL of nuclease-free water, and 2.5 µL of extracted RNA. RNA was denatured at 95°C for 5 min and then cooled down in ice for 2 min before adding to the PCR plate. The thermal profile consisted of an initial reverse transcription step at 45°C for 10 min, denaturation at 95°C for 10 min, followed by 40 cycles of denaturation at 95°C for 15 s and annealing at 53°C for 45 s. A cycle threshold (Ct) value <40 indicated positive AHSV detections.

#### Calibration and Receiver Operating Curves

Ten-fold serial dilutions of viral DNA extracted from blood taken from a healthy horse determined the dynamic and linear ranges of the AHSV-1 DIVA assay. For the vaccine strain, we used 10-fold serial dilutions in each of the AHSV-1 DIVA assay for specificity tests and tested each dilution over 3 separate runs for all assays. We generated the standard calibration curves by plotting Ct values against the logarithm of starting DNA quantities and derived slope readings from the best-fit trendline.

We used the receiver operating curve (ROC) analysis as an estimation and visual evaluation of the sensitivity and specificity performances of quantitative PCR assays. The area under the ROC curve (AUC) can also be regarded as a cumulative quality indicator for a diagnostic assay (*26*). Here, we used the pROC package (*27*) in R software (The R Project for Statistical Computing, https://www.r-project.org) to plot the ROC curves and compute the AUC values.

## Results 

**Figure 2:**
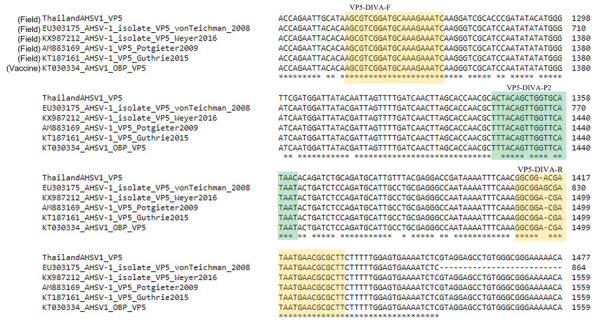
Nucleotide sequence alignment of the VP5 gene for the OBP vaccine AHSV-1 strain (GenBank accession no. KT030334), Thailand AHSV-1 field isolate (accession no. MT711962), and AHSV-1 field isolate sequences from earlier studies (accession nos. EU303175, KX987212, AM883169 and KT187161) at the 1321–1559 region (numbering according to the OBP AHSV-1 isolate), by multiple sequence alignment tool in Clustal Omega (https://www.ebi.ac.uk/Tools/msa/clustalo). Yellow indicates the the forward and reverse primer binding regions and green the probe-binding region (VP5-DIVA-P1 or P2). AHSV, African horse sickness virus; DIVA, Differentiating Infected from Vaccinated Animals; OBP, Onderstepoort Biologic Products (https://www.obpvaccines.co.za); VP, viral protein.

At the time of the outbreak in 2020, the causative strain was identified as AHSV-1 by both rRT-PCR targeting the VP7 and VP2 genes (*9*) and high-throughput sequencing (*7*,*9*), and contingency measures were implemented. The 2 rRT-PCRs we designed aimed to differentiate between clinically affected and vaccinated horses for outbreak identification and management. In silico analysis showed that the DIVA probes were specific to the AHSV-1 outbreak strain and vaccine strain and not to AHSV-3 or AHSV-4 in the trivalent vaccine (Appendix Figure 3). The VP5-DIVA-P1 probe designed for detecting the outbreak AHSV-1 strain was unable to align fully to the vaccine KT030334_AHSV1_OBP strain, whereas the same probe showed a high degree of specific alignment to the Thailand AHSV-1 sequence (Appendix Figure 1). Conversely, the VP5-DIVA-P2 probe was aligned specifically to the AHSV-1 vaccine strain but not to the outbreak AHSV-1 strain (Appendix Figure 2). In addition, the DIVA primers and probes were aligned to selected AHSV-1 sequences retrieved from GenBank, whereas the DIVA-1 probe was specific to the Thailand outbreak strain for which it was designed in this study and the DIVA-2 probe was also distinctly specific for the VP5 gene identified from vaccinated horses reported elsewhere (*18*,*28*,*29*) (Figure 2).

Using the initial batch samples that were submitted for testing (*9*), we performed 2 separate AHSV-1 DIVA rRT-PCRs to demonstrate the specificities of the primers and probes in vitro. AHSV-1 RNA was detected in 7 horse samples when using the primers with VP5-DIVA-P1 probe but not with the primers with VP5-DIVA-P2-vac probe (Table 3). On the contrary, AHSV-1 RNA was detected in AHSV-1 OBP vaccine samples only when VP5-DIVA-P2-vac probe was used (Table 3). These data have demonstrated an accuracy (100%) for both AHSV DIVA rRT-PCR methods among the small number of samples.

**Table 3:** Evaluation of AHSV-1 DIVA rRT-PCR using horse and vaccine samples from Thailand*

No.	Sample ID	Ct value by target gene (reference)			
		VP7 AHSV rRT-PCR (*20*)	VP2 AHSV-1 serotyping rRT-PCR (*22*)	VP5 (this study)	
				AHSV-1 DIVA rRT-PCR-1	AHSV-1 DIVA rRT-PCR 2
Testing of probes with initial batch samples reported in (*9*)					
1	110983/63	17.98	17.02	17.57	Undetermined
2	111495/63	22.61	20.72	22.06	Undetermined
3	111406/63	20.88	19.19	20.04	Undetermined
4	111146/63	27.49	22.70	24.69	Undetermined
5	112080/63	17.96	16.37	16.72	Undetermined
6	111789/63	19.86	18.31	18.24	Undetermined
7	111367/63	20.67	18.78	20.29	Undetermined
8	AHSV-1 OBP VACCINE 10^−3^	24.52	24.17	Undetermined	27.65
9	AHSV-1 OBP VACCINE 10^−4^	27.56	27.90	Undetermined	30.54
10	AHSV-1 OBP VACCINE 10^−5^	31.81	32.15	Undetermined	34.78
11	AHSV-1 RSArah1/03	15.21	15.38	Undetermined	17.97
Validation testing of probes with samples from clinically affected horses in this study†					
1	111146/63	18.04	17.40	18.25	Undetermined
2	111147/63-5	17.12	16.89	17.32	Undetermined
3	111147/63-19	21.30	20.12	20.14	Undetermined
4	111147/63-20	26.56	27.30	28.57	Undetermined
5	111147/63-21	23.05	22.98	23.81	Undetermined
6	111147/63-22	30.15	29.80	31.47	Undetermined
7	111162/63-A	21.67	22.75	23.16	Undetermined
8	111162/63-B	22.40	22.06	22.23	Undetermined
9	111164/63-1	28.60	28.39	28.45	Undetermined
10	111164/63-4	26.85	27.36	27.72	Undetermined
11	111367/63-B	16.44	17.69	17.51	Undetermined
12	111406/63-A	24.12	23.97	23.82	Undetermined
13	111406/63-B	18.34	18.09	18.26	Undetermined
14	111496/63	20.84	22.19	22.36	Undetermined
15	111790/63	23.62	23.83	23.88	Undetermined
16	112080/63	21.04	21.12	21.23	Undetermined
17	112590/63	26.71	29.12	29.04	Undetermined
18	112594/63	25.59	22.34	22.28	Undetermined
19	112680/63	23.62	22.84	22.50	Undetermined
20	113308/63	25.20	18.98	19.15	Undetermined
21	113480/63	25.52	26.15	25.97	Undetermined
22	113481/63	27.01	20.59	20.45	Undetermined
23	113489/63	24.58	26.19	25.94	Undetermined
24	113561/63-1	20.79	19.12	18.93	Undetermined
25	113561/63-3	33.45	19.66	19.34	Undetermined
26	113869/63	27.49	22.18	21.99	Undetermined
27	113870/63-11	19.68	35.29	34.38	Undetermined
28	113870/63-5	27.43	28.98	28.70	Undetermined
29	113871/63-N	27.32	31.34	32.02	Undetermined
30	113871/63-5140	28.32	21.76	21.31	Undetermined
31	113908/63	26.16	23.53	23.45	Undetermined
Validation testing of probes with samples from vaccinated horses in this study					
1	116187/63	27.48	34.19	Undetermined	35.62
2	122045/63	31.60	33.12	Undetermined	35.37
3	135560/63	38.59	36.71	Undetermined	38.79
4	137720/63	34.29	33.36	Undetermined	35.32
5	118696/63	32.31	32.79	Undetermined	34.37
6	120865/63	32.23	34.03	Undetermined	35.16
7	136979/63	32.33	31.40	Undetermined	32.92
8	121673/63-A	33.24	31.16	Undetermined	33.22
9	121673/63-B	33.89	32.81	Undetermined	34.72
10	121673/63-C	34.37	32.39	Undetermined	33.85
11	124916/63-A	36.45	35.11	Undetermined	36.53
12	124916/63-B	36.45	33.69	Undetermined	35.51

*Samples that were initially submitted for testing (*9*) were used in the first round of designed assay optimization. Subsequently, EDTA blood and tissues samples were taken from clinically affected and vaccinated horses for assay validation. Ct values were used for classification of positive AHSV detections. AHSV, African horse sickness virus; Ct, cycle threshold; DIVA, Differentiating Infected from Vaccinated Animals; ID, identification; rRT-PCR, real-time reverse transcription PCR; VP, viral protein. †AHSV-1 serotyping rRT-PCR was not carried out for the clinically affected and vaccinated horse samples used in the confirmatory rRT-PCR reactions to test the DIVA assays. Cycle threshold values not registered after 40 cycles were reported as undetermined.

To substantiate the observations that the designed primer pairs are indeed specific and sensitive enough to differentiate between the vaccine and wild strain of AHSV-1, we collected more blood and tissue samples from both the prevaccinated and postvaccinated animals from horses in the Thailand outbreak area. Using these samples, we again observed that AHSV-infected (i.e., clinically affected) and vaccinated horses could be differentiated by using the VP5-DIVA-P1 and VP5-DIVA-P2-vac assays designed in this study with an accuracy of 100% (Table 3). The Ct values obtained from 3 RT-PCR runs using the VP5-DIVA-P1 on clinically affected horses’ samples were 17.32–34.38, whereas those obtained using the VP5-DIVA-P2-vac on the vaccinated horses’ samples were 33.22–38.79. No cross-amplification of the samples with the alternate probe (i.e., clinically affected samples amplified with the VP5-DIVA-P2-vac probe and vice versa) resulted from the quantitative PCR run. These data suggest this VP5 gene–based DIVA assay can readily and accurately differentiate the vaccine strain from the outbreak strain with reproducibility. The samples were also confirmed by VP7- and VP2-targeted rRT-PCR for AHSV (Table 3). Two infected horses, 112080/63 and 110983/63, with high viral load (Ct values 17.96 and 17.98 by VP7 rRT-PCR) tested negative using DIVA rRT-PCR 2 (Table 3). 

We also examined cross-reactivity between 2 DIVA PCRs. Because high-dose LAV was unavailable in our laboratory, we used a cell culture isolate AHSV-1 South Africa RSArah1/03 (supplied by the Pirbright Institute), which is highly similar to the OBP LAV vaccine strain (99.73% of genome similarity). The isolate produced signal only with DIVA rRT-PCR 2 (Ct 17.97) and not DIVA rRT-PCR 1 (Table 3).

We further tested the analytical sensitivity of the DIVA assay by testing 10-fold serial dilutions of 1 AHSV-1 outbreak sample (AUC = 0.943, slope = −3.89) (Table 4) as well as the vaccine sample (AUC = 0.900; slope = −3.49) (Table 4). The analytical sensitivity of the DIVA assays was comparable to the serotyping assay and 10-fold less than the AHSV VP7 rRT-PCR (Table 4). However, compared with the VP2 sequencing–based DIVA methodology, the DIVA rRT-PCR increases analytical sensitivity, reduces cost, and shortens turnaround time. The detection rate of subclinical infections could also be substantially increased in the period leading up to a potential outbreak.

**Table 4:** Comparison of assay sensitivity among different AHSV assays*

Sample	Method	Limit of detection	AUC	Slope
AHSV-1 Thailand 110983/63	AHSV VP7 rRT-PCR	[10^−5^]	0.943	−3.89
	AHSV-1 VP2 Serotyping rRT-PCR	[10^−4^]		
	AHSV-1 VP5 DIVA rRT-PCR 1	[10^−4^]		
AHSV-1 OBP LAV	AHSV VP7 rRT-PCR	[10^−6^]	0.900	−3.49
	AHSV-1 VP2 Serotyping rRT-PCR	[10^−5^]		
	AHSV-1 VP5 DIVA rRT-PCR 2	[10^−5^]		

*The limit of detection was reported from the lowest observed amount of DNA amplified below the Ct <40 threshold. The AUC and slope values derived from the calibration curve are also listed. AHSV, African horse sickness virus; AUC, area under the receiver operating curve; Ct, cycle threshold; DIVA, Differentiating Infected from Vaccinated Animals; OBP LAV, Onderstepoort Biologic Products (https://www.obpvaccines.co.za) live attenuated vaccine; rRT-PCR, real-time reverse transcription PCR; VP, viral protein.

## Discussion

Genetic recombination is a mechanism and strategy observed in most viruses for adaptation and survival. The introduction of polyvalent LAV could serve as a pool of readily available genetic segments for comultiplication and reassortment within the host (*18*,*28*,*29*). Immunized horses can still be infected by AHS, especially by the AHSV-1-LAV within the trivariant AHSV-LAV formulation (*30*). Manole et al. (*31*) also demonstrated by high-resolution imaging that the physical structure of certain serotypes of AHSV could undergo distortion in the event of host–virus interaction. However, the exact reversion-to-virulence mutants and reassortants in the presence of AHSV field strains are not well understood. Despite the considerations of using monovalent AHSV vaccines to confer immunity to horses (*32*,*33*), the protection coverage is not as good as the 2-dose polyvalent OBP LAV used in this outbreak (*34*).

After the Thailand outbreak in which AHSV-1 was the causative agent, efforts at disease outbreak management and animal protection were focused on this serotype. The AHSV-1 DIVA assay described in this study only applies to the OBP LAV currently used in the vaccination campaign in Thailand. The method should be modified if a different AHSV strain or a different vaccine is used. Recombinant vaccine with VP5 gene from AHSV strains other than the strain used by OBP might render this assay ineffective. Alternative candidate DIVA regions or vaccine candidates can also be examined and evaluated using the approach described here. Other molecular strategies deploying the DIVA strategy can be explored, such as high-resolution melting analysis to identify the variations between the vaccine and wild-type strains.

Early detection and differentiation of the AHSV causative agent is vital for identifying and managing at-risk horses. Complementing the earlier study by Toh et al. (*9*), our rapid development of a DIVA assay relevant to the outbreak at hand was useful for surveillance and control of the outbreak to prevent wider onwards transmission of the disease. This DIVA strategy will be useful for a regional vaccination strategy in southeast Asia, where the wide distribution of the *Culicoides* spp. vector poses a biorisk of AHSV incursion. The DIVA assay also enables identification of vaccine breakthroughs. The next step is to validate the DIVA assay with field samples from horses worldwide, particularly for horses vaccinated using the OBP LAV. DIVA strategy could support the surveillance of AHSV at the genomic level to identify recombinants, reassortants, or mutants among vaccinated equids. This method to rapidly characterize the affecting field strain and develop a differentiating method should be applied in future outbreaks to determine the epidemiology of AHSV.

Appendix Additional information about development of Differentiating Infected from Vaccinated Animals (DIVA) real-time PCR for African horse sickness virus serotype 1
