# Supplementary material for: Development of Differentiating Infected from Vaccinated Animals (DIVA) Real-Time PCR for African Horse Sickness Virus Serotype 1
Source: Emerg Infect Dis. 2022 Dec;28(12):2446–54. doi: 10.3201/eid2812.220594 (PMC9707579; doi:10.3201/eid2812.220594)
Supplement: Appendix — Additional information about development of Differentiating Infected from Vaccinated Animals (DIVA) real-time PCR for African horse sickness virus serotype 1 [file 22-0594-Techapp-s1.pdf]

Article DOI: https://doi.org/10.3201/eid2812.220594

Development of Differentiating Infected from Vaccinated Animals (DIVA) Real-Time PCR for African Horse Sickness Virus Serotype 1

Appendix

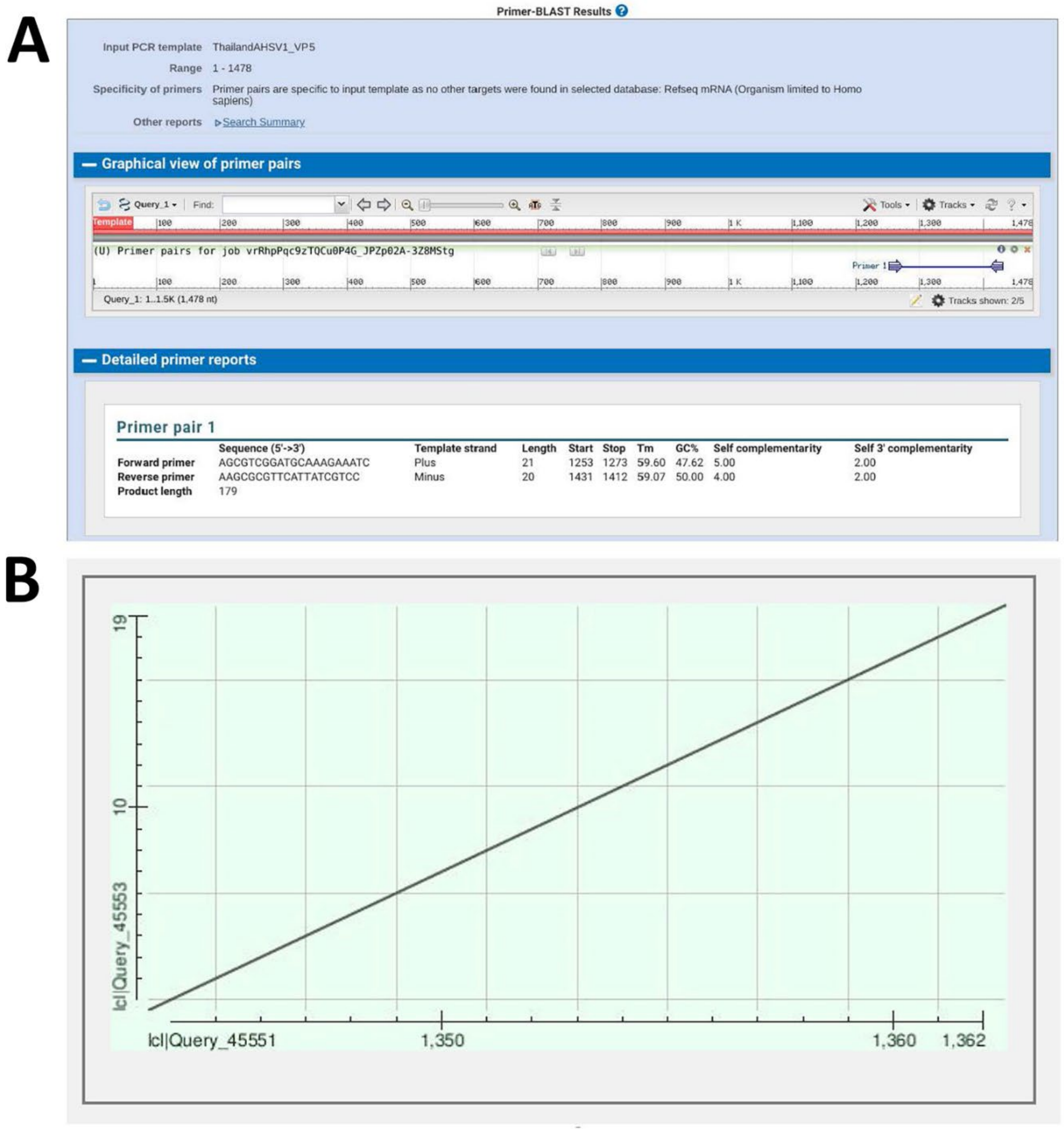

C

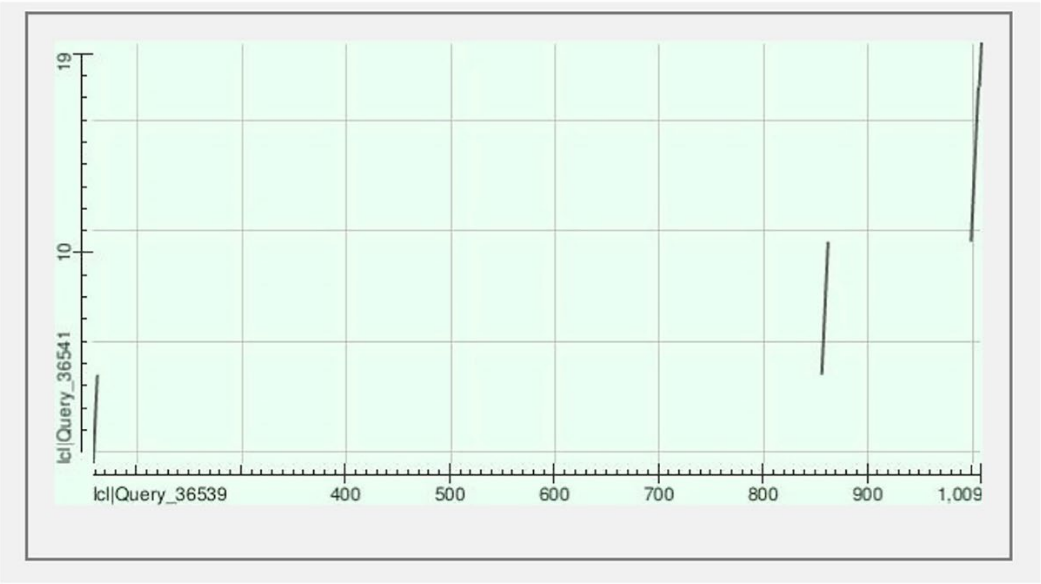

Appendix Figure 1. Summary of alignment plots of primers or probes to the vaccine (AHSV-1, AHSV-3 and AHSV-4) and outbreak (ThailandAHSV1) strains. These are made available from the Primer-BLAST and BLAST Global Alignment (dot plots) programs. A) Primer VP5-DIVA-P1 vs ThailandAHSV1 by Primer-BLAST; B) Primer VP5-DIVA-P1 vs ThailandAHSV1 by BLAST Global Alignment by the Needleman-Wunsch algorithm; C) VP5-DIVA-P2 vs ThailandAHSV1 by BLAST Global Alignment by the Needleman-Wunsch algorithm.

A

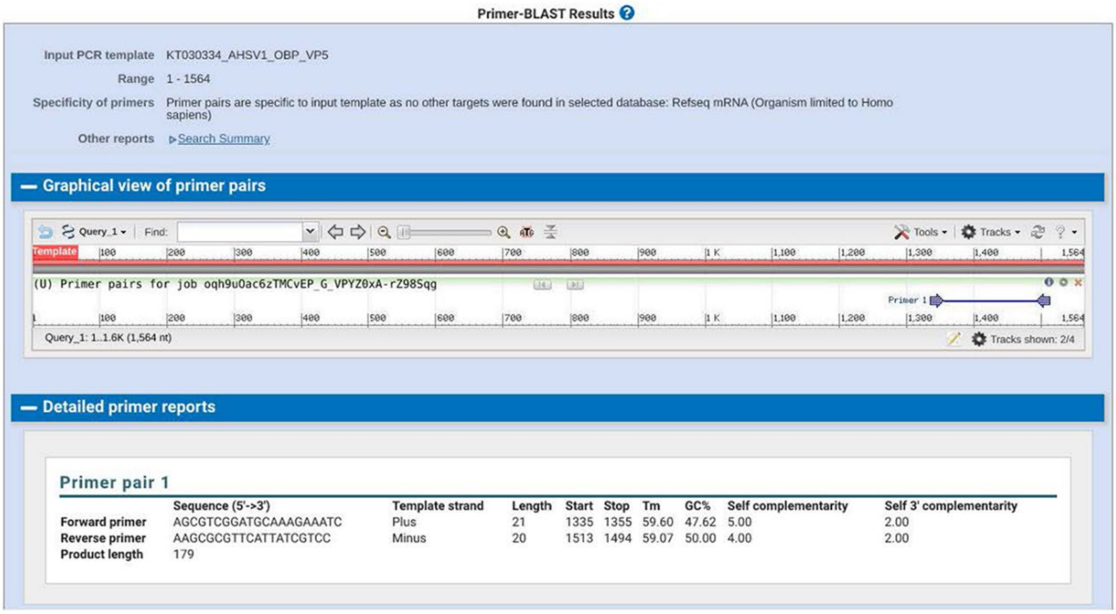
Primer-BLAST Results
Input PCR template KT030334_AHSV1_OBP_VP5
Range 1 - 1564
Specificity of primers Primer pairs are specific to input template as no other targets were found in selected database: Refseq mRNA (Organism limited to Homo sapiens)
Other reports Search Summary
Graphical view of primer pairs
Query_1
Find:
Tools
Tracks
Template
(U) Primer pairs for job oqh9uOac6zTMCvEP_G_VPYZ0xA-rZ98Sqg
Primer 1
Query_1: 1..1.6K (1,564 nt)
Tracks shown: 2/4
Detailed primer reports
Primer pair 1
Sequence (5'->3') Template strand Length Start Stop Tm GC% Self complementarity Self 3' complementarity
Forward primer AGCGTCGGATGCAAAGAAATC Plus 21 1335 1355 59.60 47.62 5.00 2.00
Reverse primer AAGCGCGTTCATTATCGTCC Minus 20 1513 1494 59.07 50.00 4.00 2.00
Product length 179

B

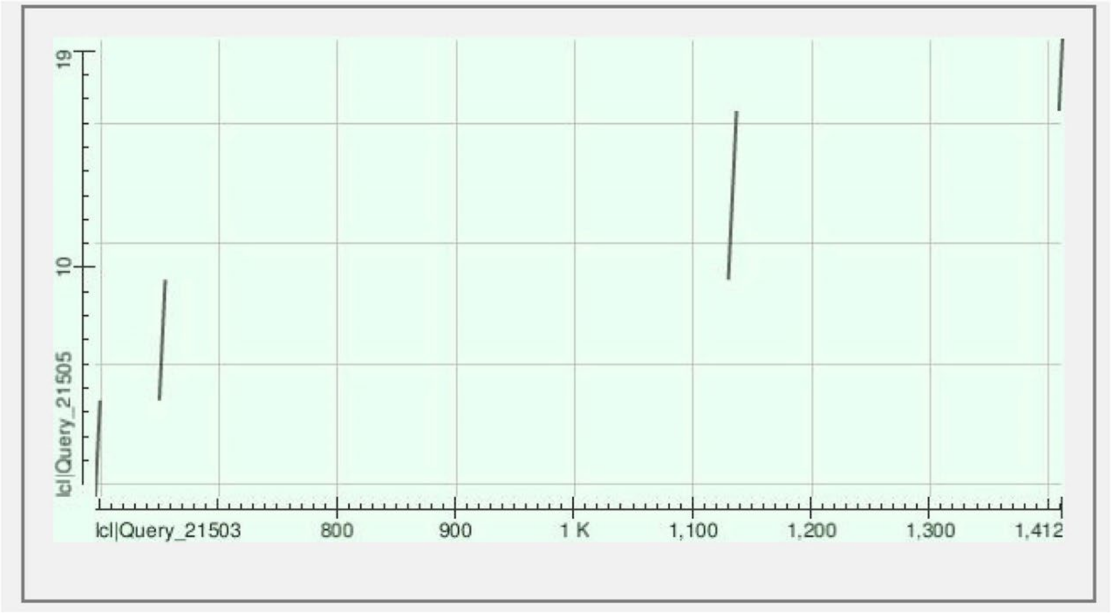
lcl|Query_21505
19
10
lcl|Query_21503
800
900
1 K
1,100
1,200
1,300
1,412

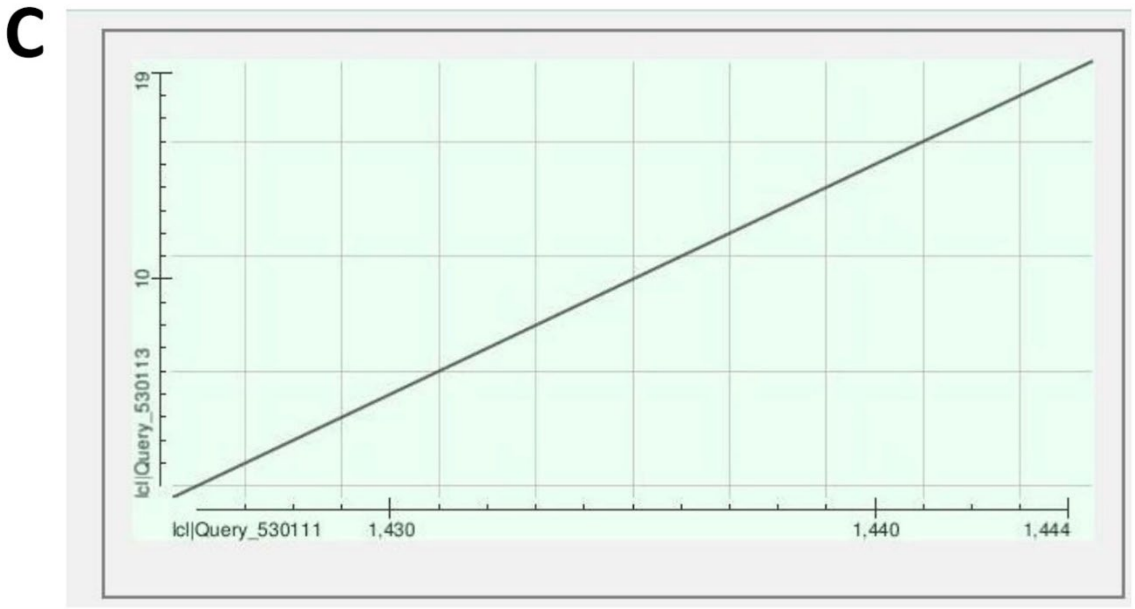

Appendix Figure 2. Summary of alignment plots of primers or probes to the vaccine (AHSV-1, AHSV-3 and AHSV-4) and outbreak (ThailandAHSV1) strains. These are made available from the Primer-BLAST and BLAST Global Alignment (dot plots) programs. A) Primer vs AHSV_1; B) VP5-DIVA-P1 vs AHSV_1 by BLAST Global Alignment by the Needleman-Wunsch algorithm; C) VP5-DIVA-P2 vs AHSV1 by BLAST Global Alignment by the Needleman-Wunsch algorithm.

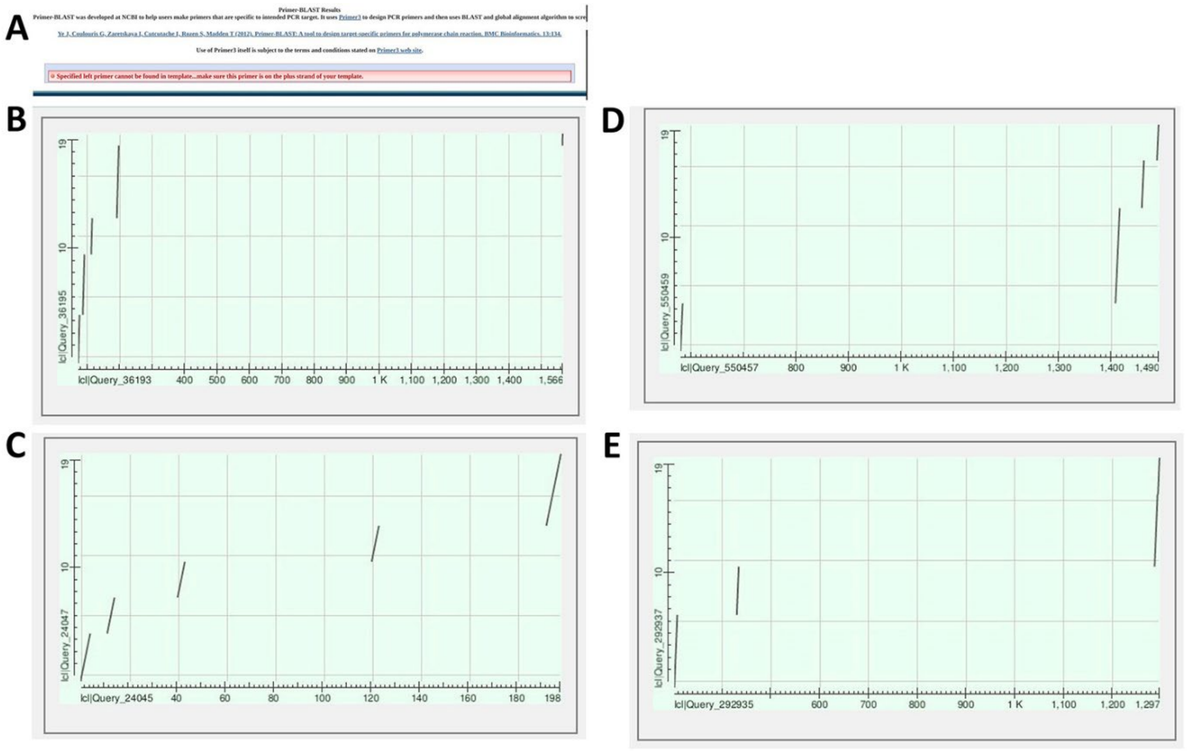

Appendix Figure 3. Summary of alignment plots of primers or probes to the vaccine (AHSV-1, AHSV-3 and AHSV-4) and outbreak (ThailandAHSV1) strains, by BLAST Global Alignment by the Needleman-Wunsch algorithm (dot plots). A) Primers vs AHSV_3 / AHSV_4; B) VP5-DIVA-1 vs AHSV_3; C) VP5-DIVA-2 vs AHSV_3; D) VP5-DIVA-1 vs AHSV_4; E) VP5-DIVA-2 vs AHSV_4.
